# Anterior thalamic nucleus stimulation protects hippocampal neurons by activating autophagy in epileptic monkeys

**DOI:** 10.18632/aging.103026

**Published:** 2020-04-08

**Authors:** Ting-Ting Du, Guanyu Zhu, Yingchuan Chen, Lin Shi, Defeng Liu, Yuye Liu, Xin Zhang, Jianguo Zhang

**Affiliations:** 1 Department of Functional Neurosurgery, Beijing Neurosurgical Institute, Capital Medical University, Beijing 100070, China; 2 Department of Neurosurgery, Beijing Tiantan Hospital, Capital Medical University, Beijing 100070, China; 3 Beijing Key Laboratory of Neurostimulation, Beijing 100070, China

**Keywords:** epilepsy, deep brain stimulation, anterior thalamic nuclei, autophagy, brain-derived neurotrophic factor

## Abstract

Deep brain stimulation of the anterior nucleus of the thalamus (ANT-DBS) is effective in treating temporal lobe epilepsy (TLE) and protects hippocampal neurons. Autophagy plays an essential role in epileptogenesis; however, the underlying effect of autophagy on ANT-DBS-mediated neuroprotection remains unclear. A monkey model of epilepsy was established by injecting kainic acid into the hippocampus and amygdala using a robot-assisted system. ANT-DBS was delivered in the chronic stage of the epileptic model and continued for 8 weeks. We found that ANT-DBS reduced the frequency of seizures and exerted neuroprotective effects via activating autophagy in hippocampal neurons. ANT-DBS increased light chain 3 (LC3) II level and co-localization of LC3 and lysosomal-associated membrane protein-1, accompanied by decreased expression of the autophagy substrate ubiquitin-binding protein p62, suggesting increased autophagosome formation. Most importantly, brain-derived neurotrophic factor (BDNF) –tropomyosin-related kinase type B (TrkB) pathway were involved in the regulation of autophagy. Both protein levels were reduced by ANT-DBS, and there was less phosphorylation of downstream regulators, extracellular signal-regulated kinase and Akt, followed by inactivation of mammalian target of rapamycin complex 1. Taken together, chronic ANT-DBS exerts neuroprotective effects on hippocampal neurons through inducing autophagy via suppressing the BDNF–TrkB pathway in a TLE monkey model.

## INTRODUCTION

Epilepsy is characterized by a predisposition to generating recurrent seizures with neurobiological, cognitive, psychological and social consequences [1]. Even if treated with the new antiepileptic drugs, approximately one-third of patients with epilepsy cannot achieve seizure control, in particular those with complex partial epilepsy [2]. Anterior nucleus of the thalamus (ANT) is now the most frequently used deep brain stimulation (DBS) targets for drug-resistant temporal lobe epilepsy (TLE) [3]. A double-blind randomized control trial revealed a median seizure reduction from baseline of 41% at one year and 69% at five years. On account of the previously demonstrated safety and efficacy of DBS, the US Food and Drug Administration (FDA) approved it for epilepsy in 2018 [4–6].

Hippocampal neuronal loss and Papez circuit reformation are the most important pathogenic features of TLE [7, 8]. It has already been found that ANT-DBS has a protective effect on the hippocampal neurons [9, 10], but the underlying mechanism still needs to be elucidated. Autophagy can attenuate inflammation and eliminate damaged mitochondria to maintain neuronal homeostasis [11, 12], which has a close relationship with epileptogenesis. Some researchers have found that knockout of *ATG7*, a regulator of autophagy, induces lethal epilepsy in mice [13]. Rapamycin, an inducer of autophagy, is effective in the tuberous sclerosis and some other type epilepsy including anoxia, kainic acid (KA) or pilocarpine induced epilepsy [14, 15]. Inhibition of the mammalian target of rapamycin complex (mTORC)1 function by rapamycin or a ketogenic diet can promote autophagy and control epilepsy [16]. One of the most important upstream inhibitors of autophagy is brain-derived neurotrophic factor (BDNF), which acts via activation of its high-affinity receptor tropomyosin-related kinase type B (TrkB). Many studies have found the BDNF level is elevated in chronic epilepsy models [17, 18]. The seizure-induced expression of neurotrophic factors might contribute to the lasting structural and functional changes underlying epileptogenesis, like mossy fiber sprouting [19–21]. BDNF is crucial for regulating the baseline autophagic activity in the brain [21]. However, the effect of autophagy on ANT-DBS-mediated neuroprotection has not been elucidated.

Several important structures in the basal ganglia and limbic system are implicated in seizure propagation, and these structures have major differences between non-human primates (NHP) and rodents [22–24]. NHP are more similar to humans. So, for better evaluation of the DBS effect, we used a KA injection primate model in this study.

We used our robot-assisted NHP ANT-DBS implantation platform and well-characterized intra-hippocampal and intra-amygdala KA epilepsy model to investigate the molecular mechanism of the neuroprotective effects of ANT-DBS [25, 26]. The most important finding was that ANT-DBS activated autophagy by inhibiting the BDNF–TrkB signaling pathway and then suppressing mTOR activation, thereby protecting hippocampal neurons against KA-induced apoptosis. Our results in this primate model of TLE, the one most readily applicable to humans, will hopefully provide insights into the neuroprotective effects of ANT-DBS in TLE patients.

## RESULTS

### Electrode positions and details of ANT-DBS

**Figure 1:**
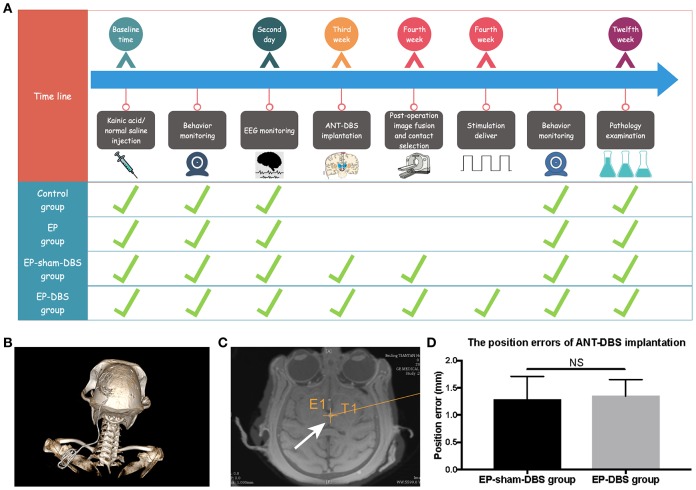
**Experimental design and the lead location.** (**A**) Twenty-four monkeys were randomly assigned to the four treatment groups, which differed with respect to manipulations. The time from the beginning of the manipulation is shown in the first line, and the green ticks indicate manipulation in each group. (**B**) 3D reconstruction of postoperative CT showed that the DBS lead was implanted through the frontoparietal skull and extended to the back. (**C**) The lead was accurately placed in the left ANT based on the merged pre-operative MR and post-operative CT (white arrow). The cross indicates the surgical planning target. (**D**) The position errors of ANT-DBS implantation between the EP-sham-DBS and EP-DBS groups were similar and ideal (n=6 in each group). NS, *P*> 0.05. Data were presented as mean ± SD.

The detail manipulation and grouping were described in MATERIALS AND METHODS Section (Figure 1A). Briefly, all monkeys were randomly assigned to control group, EP group, EP-sham-DBS group and EP-DBS group. TLE monkey models were established in EP group, EP-sham-DBS group and EP-DBS group. And ANT-DBS was implanted in EP-sham-DBS group and EP-DBS group, however, only the TLE monkeys in EP-DBS group received stimulation. DBS electrode positions were evaluated by image fusion in the robot workstation. The position error in the EP-sham-DBS and EP-DBS groups was 1.28 ± 0.42 and 1.35 ± 0.30 mm, respectively, without significant difference between the two groups (*P* > 0.05) (Figure 1B–1D), which was believed to be the acceptable position error. The contacts within the ANT were selected for stimulation based on the fusion images, delivering a pulse stimulation of 1.5 V, 90 μs and 150 Hz (Contact-, IPG+).

### ANT-DBS reduced seizure frequency in epileptic monkeys

**Figure 2:**
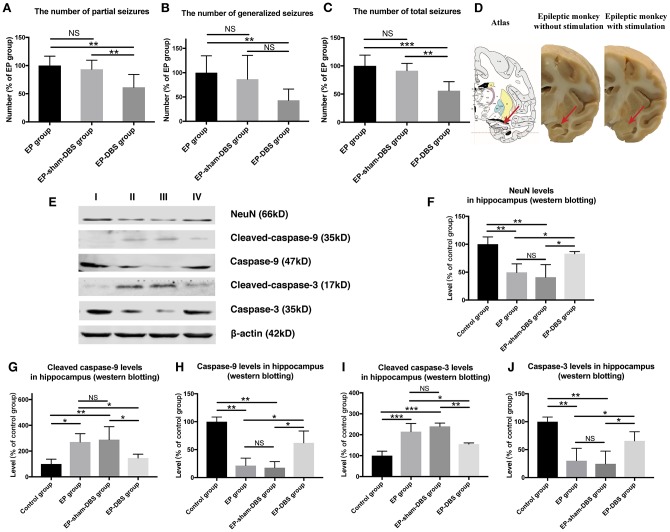
**ANT-DBS reduced seizures frequency and relieved hippocampal neurons apoptosis in the epileptic monkeys.** (**A**–**C**) Numbers of seizures in the different groups. The numbers of partial and total seizures were reduced by ANT-DBS, compared with the EP and EP-sham-DBS groups. Although there was no significant difference in the number of generalized seizures between EP-sham-DBS and EP-DBS groups (*P* > 0.05), there was still a trend of reduction. (n=6 in each group) (**D**) Morphology of hippocampus in monkeys receiving ANT-DBS was not affected and there was no obvious atrophy. Red arrows indicate the location of the hippocampus in the atlas of the rhesus monkey brain. (**E**) Analysis of NeuN, cleaved-caspase-3 caspase-3, cleaved-caspase-9 and caspase-9 by western blotting. (**F**–**J**) There were marked decrease in NeuN level and increase in cleaved-caspase-3 and cleaved-caspase-9 levels in EP and EP-sham-DBS groups. Increased NeuN and reduced cleaved-caspase-3 and cleaved-caspase-9 levels were detected after ANT-DBS. (n=3 in each group) *P< 0.05; **P< 0.01; ***P< 0.001; NS, *P*> 0.05. Data were presented as mean ± SD. I, control group; II, EP group; III, EP-sham-DBS group; IV, EP-DBS group.

Status epilepticus and spontaneous recurrent seizures were induced in animals that received KA injection. No abnormal or epileptic behavior was recorded in the control group, whereas abnormal EEG was recorded in all animals that received KA injection, which was confirmed by our previous study [25]. Epileptic behavior was monitored and recorded in all groups at the chronic stage (1 month after KA or normal saline injection). A similar number of partial seizure, generalized seizure and total seizure were obtained in EP-sham-DBS and EP group (all *P* > 0.05), however, the number of partial seizures and total seizures were reduced by ANT-DBS (partial seizures: all *P* < 0.01, vs EP and EP-sham-DBS group; total seizures: *P* < 0.001, vs EP group; *P* < 0.01, vs EP-sham-DBS group). Although there was no significant difference in the number of generalized seizures between EP-sham-DBS and EP-DBS groups (*P* > 0.05), there was still a trend of reduction (Figure 2A–2C).

### ANT-DBS relieved apoptosis and protected neurons in epileptic monkeys

Hippocampal atrophy was obvious in the EP and EP-sham-DBS groups. Hippocampal atrophy, however, did not occur in ANT-DBS treated animals (Figure 2D). Numerous studies have revealed that hippocampal atrophy is mainly caused by neuronal loss, so we evaluated the protein level of NeuN, a specific neuron marker, and the level of cleaved-caspase-3 and cleaved-caspase-9, markers of apoptosis. Also, the caspase-3 and caspase-9 were evaluated. Compared with the control group, there was a significant decrease in NeuN (F_(3,8)_= 10.007, *P* <0.01) and an increase in cleaved-caspase-3 (F_(3,8)_= 20.735, *P* < 0.001) and cleaved-caspase-9 (F_(3,8)_= 6.148, *P* < 0.05) in the EP and EP-sham-DBS groups, indicating severe neuronal loss and apoptosis. This phenomenon was alleviated after ANT stimulation in the EP-DBS group, with enhanced NeuN, and reduced cleaved-caspase-3 and cleaved-caspase-9 level in comparison with the EP and EP-sham-DBS groups. Meantime, it is found that the decreased caspase-3 (F_(3,8)_= 10.704, *P* <0.01) and caspase-9 (F_(3,8)_= 20.096 *P* < 0.001) were reversed by ANT-DBS (Figure 2E–2J).

**Figure 3:**
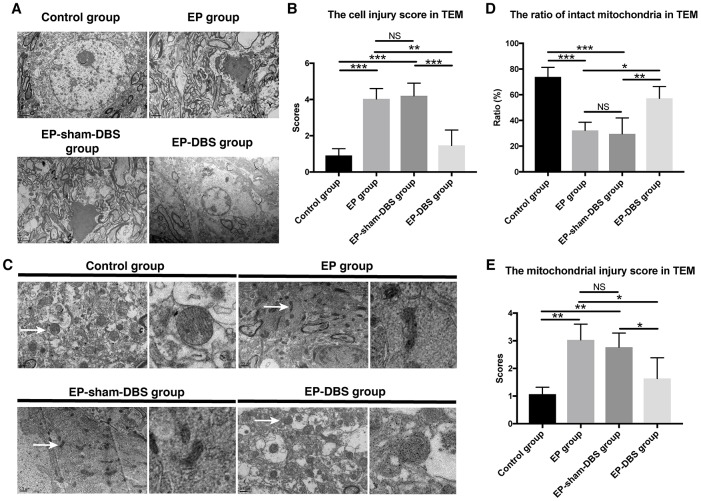
**ANT-DBS protected hippocampal neurons of the epileptic monkeys.** (**A**) Neuronal ultrastructure by TEM. The injury was severe in the hippocampus of EP and EP-sham-DBS groups. The injury, however, was relieved by ANT-DBS. Magnification: ×1000. (**B**) Higher injury grades were obtained in the EP and EP-sham-DBS groups, and this grade was alleviated by ANT-DBS. (n=3 in each group; in each monkey, twenty cells were randomly selected, and the average score for each monkey was recorded) (**C**) Morphology of mitochondria (white arrow) by TEM. Normal or slightly abnormal morphology of mitochondria was seen in the control and EP-DBS groups. The EP and EP-sham-DBS groups showed swelling of the mitochondrial matrix, sometimes with disrupted membrane integrity. Magnification: ×3000. The right column shows a closer view of mitochondria. (**D**) The number of intact mitochondria in the EP and EP-sham-DBS groups was lower than that in the control and EP-DBS groups. (n=3 in each group; in each monkey, twenty fields were randomly selected, and the average number for each monkey was recorded) (**E**) The grade of mitochondrial injury was higher in the EP and EP-sham-DBS groups, and reversed by ANT-DBS (n=3 in each group; in each monkey, twenty cells were randomly selected, and the average grade for each monkey was recorded). *P< 0.05; **P< 0.01; ***P< 0.001; NS, *P*> 0.05. Data were presented as mean ± SD.

We also evaluated the cell injury and the mitochondrial injury follow the criteria using TEM (Tables 1 and 2). In the EP and EP-sham-DBS groups, cell injury was severe in the hippocampus with numerous fragments from disintegrated organelles. The neurons were predominantly pyknotic, with or without complete cell membranes. The neuronal nuclei were either deformed or ruptured, with extensive karyorrhexis and karyolysis. However, EP-DBS group animals had neuronal injury no different from control group, presenting cellular integrity with some swelling of organelles in some neurons (Figure 3A). According to the criteria, the injury scores were elevated in the EP and EP-sham-DBS groups compared with the control group, and were reduced in the EP-DBS group (F_(3,8)_= 20.837, *P* < 0.001) (Figure 3B).

**Table 1:** Scoring system for neuronal injury by transmission electron microscopy.

**Score**	**Injury observed in TEM**
0	Basically normal.
1	Slightly injured, focally distended ER, condensed or swollen M, fovea on karyolemma.
2	Mildly injured, general swelling of organelles, clearly decreased cytoplasmic electron density, major depression of karyolemma.
3	Moderately injured, severe swelling of the entire cell, formation of cytoplasmic vacuoles or blebs, evident cell shrinkage, transparent cytoplasm.
4	Severely injured, pyknotic cells with deformed, but integrated cell membrane and karyolemma, apoptosis.
5	Near death, pyknotic cells with trace of karyorrhexis or karyolysis, disruption of cell membrane, rupture of karyolemma, disintegration of organelles, apoptotic bodies.

M: mitochondria. ER: endoplasmic reticulum. Minimal increment: 0.5

**Table 2:** Scoring system for mitochondrial injury by transmission electron micrography.

**Score**	**Observation**
0	Normal mitochondria (mitochondria appeared highly dense with well-organized cristae)
1	Early swelling as manifested by early clearing of matrix density and separation of cristae (a large amorphous matrix density and a linear density are present)
2	More marked swelling as manifested by further clearing of matrix density and separation of cristae
3	More extensive mitochondrial swelling with disruption of cristae
4	Severe mitochondrial swelling with disruption of cristae and rupture of inner and outer mitochondrial membranes

Marked mitochondrial ultrastructural injury was observed in the EP and EP-sham-DBS groups, with significant swelling of the mitochondrial matrix. In some instances, mitochondrial swelling was accompanied by disruption of membrane integrity. In contrast, mitochondria in the EP-DBS group had normal morphology, with no or only slight evidence of swelling, outer membrane breakage, or intracristal dilation, which was similar to that in the control group (Figure 3C). According to the criteria, grades 0 and 1 were considered as intact mitochondria, and less intact mitochondria were found in the epileptic model, whereas, ratio of intact mitochondria was enhanced in monkeys that received ANT-DBS (F_(3,8)_= 16.116, *P* < 0.001) (Figure 3D). The mean mitochondrial injury grade was further evaluated in term of the criteria mentioned above, and showed a similar tendency (F_(3,8)_= 8.567, *P* < 0.01) (Figure 3E).

ANT-DBS inhibited BDNF–TrkB signaling, and subsequently reduced Akt and ERK phosphorylation in the hippocampus of epileptic monkeys

**Figure 4:**
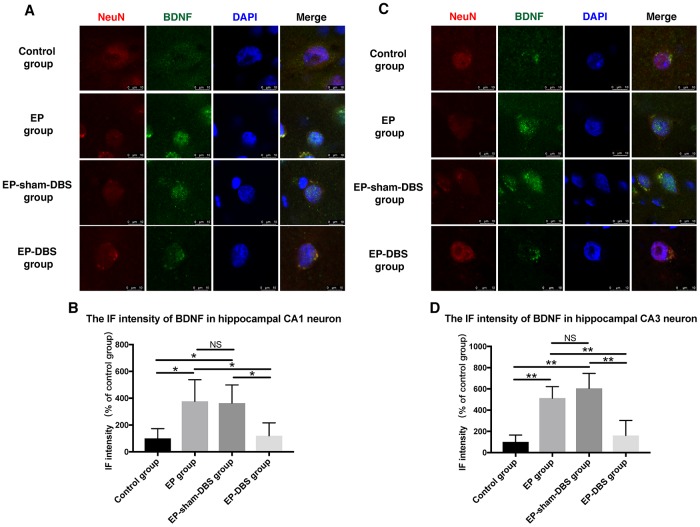
**ANT-DBS reduced BDNF expression in the hippocampal neurons of epileptic monkeys.** Immunofluorescence staining of BDNF and NeuN in hippocampal CA1 (**A**) and CA3 (**C**) neurons. NeuN is a specific neuronal marker. BDNF expression was elevated in EP and EP-sham-DBS groups in hippocampal CA1 and CA3 neurons and reduced by ANT-DBS. Immunofluorescent intensity of BDNF in hippocampal CA1 (**B**) and CA3 (**D**) neurons was quantified. (n=3 in each group; in each monkey, a total of thirty cells in CA1 or CA3, the average immunofluorescent intensity of these cells was recorded) *P < 0.05; **P < 0.01; NS, *P* > 0.05. Data were presented as mean ± SD.

**Figure 5:**
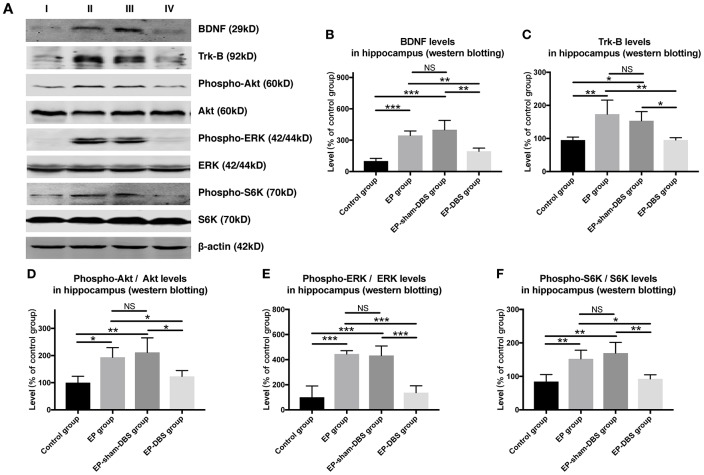
**ANT-DBS inhibited BDNF-TrkB pathway and its downstream regulator in the hippocampus of epileptic monkeys.** (**A**) Analysis of BDNF, TrkB, p-ERK, ERK, p-Akt, Akt, p-S6K and S6K by western blotting. (**B**–**C**) In EP and EP-sham-DBS groups, BDNF and TrkB levels were elevated compared with the control group. With ANT-DBS, less BDNF and TrkB were observed. (n=3 in each group) (**D**–**F**) In the epileptic animal model, the ratio of p-Akt/Akt, p-ERK/ERK and p-S6K/S6K was enhanced in the hippocampus, and reversed by ANT-DBS. (n=3 in each group) *P < 0.05; **P < 0.01; ***P < 0.001; NS, *P* > 0.05. Data were presented as mean ± SD. I, control group; II, EP group; III, EP-sham-DBS group; IV, EP-DBS group.

Immunofluorescence staining of BDNF and NeuN was carried to evaluate BDNF expression in neurons. Significantly more BDNF was observed in hippocampal CA1 (F_(3,8)_= 4.620, *P* < 0.05) and CA3 (F_(3,8)_= 13.564, *P* < 0.01) neurons in the EP and EP-sham-DBS groups, and less was detected in the EP-DBS group (Figure 4). BDNF–TrkB plays an essential role in regulating autophagy via the Akt and ERK pathway [21]. In the EP and EP-sham-DBS groups, BDNF (F_(3,8)_= 19.242, *P* < 0.001) and TrkB (F_(3,8)_= 7.107, *P* < 0.05) levels were elevated compared with the control group. With ANT-DBS, notably less BDNF and TrkB were detected (Figure 5A–5C).

Akt and ERK are the most important downstream regulators of the BDNF–TrkB signaling pathway, and activation of the pathway is shown by phosphorylation of Akt and ERK (p-Akt and p-ERK). In the epileptic animal model, p-Akt (F_(3,8)_= 6.729, *P* < 0.05) and p-ERK (F_(3,8)_= 23.642, *P* < 0.001) were enhanced in the hippocampus; however, p-Akt and p-ERK levels were decreased by ANT-DBS, and showed a similar trend to that shown by BDNF–TrkB (Figure 5A, 5D and 5E).

Phosphorylation of S6K (p-S6K) has been used as a marker of activation by mTORC1 and is positively correlated with the inhibition of autophagy. We found that ANT-DBS reversed the increased p-S6K (F_(3,8)_= 9.635, *P* < 0.01) level in epileptic monkeys (Figure 5A and 5F). No significant difference in above was detected between the EP and EP-sham-DBS groups.

### ANT-DBS activated autophagy in the hippocampus of epileptic monkeys

**Figure 6:**
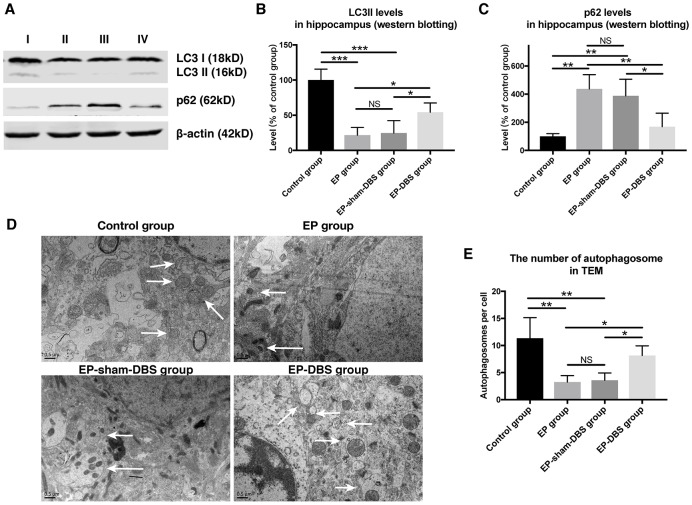
**ANT-DBS activated autophagy in the hippocampus of epileptic monkeys.** (**A**) Analysis of LC3II and p62 by western blotting. (**B**) Levels of LC3II were decreased in EP and EP-sham-DBS groups. ANT-DBS normalized LC3II level in the EP-DBS group. (n=3 in each group) (**C**) In the EP and EP-sham-DBS groups, p62 levels were increased, and decreased by ANT-DBS. (n=3 in each group) (**D**) Autophagosome observed by TEM. The left column shows the morphology of the autophagosome by TEM. In the other columns, the autophagosomes (white arrow) are shown around the nucleus. (**E**) The number of autophagosomes was counted in each view. A reduced number of autophagosomes were observed in the EP and EP-sham-DBS groups compared with the control group, which was increased by ANT-DBS. (n=3 in each group; in each monkey, twenty cells were randomly selected, and the average number of autophagosome for each monkey was recorded) *P < 0.05; **P < 0.01; ***P < 0.001; NS, *P* > 0.05. Data were presented as mean ± SD. I, control group; II, EP group; III, EP-sham-DBS group; IV, EP-DBS group.

In the formation of autophagosomes, the cytosolic form of LC3I is converted to the lipid-conjugated LC3II form (a marker of autophagosome). To investigate the effect of ANT-DBS on autophagy induction, LC3II protein level was assessed. ANT-DBS resulted in up-regulation of LC3II compared to those in the EP and EP-sham-DBS groups (F_(3,8)_= 18.766, *P* < 0.001) (Figure 6A and 6B). P62 is a substrate of autophagy and its level was determined in the different groups. The elevation of P62 protein level in the EP and EP-sham-DBS groups was reduced by ANT-DBS (F_(3,8)_= 9.527, *P* < 0.01) (Figure 6A and 6C). These results indicated that autophagosome formation was induced by ANT-DBS in epileptic monkeys.

The number of autophagosomes was also measured by TEM. Fewer autophagosomes were observed in the EP and EP-sham-DBS groups compared with the control group. Nevertheless, ANT-DBS induced a marked increase in the number of autophagosomes (F_(3,8)_= 8.636, *P* < 0.01) (Figure 6D and 6E).

**Figure 7:**
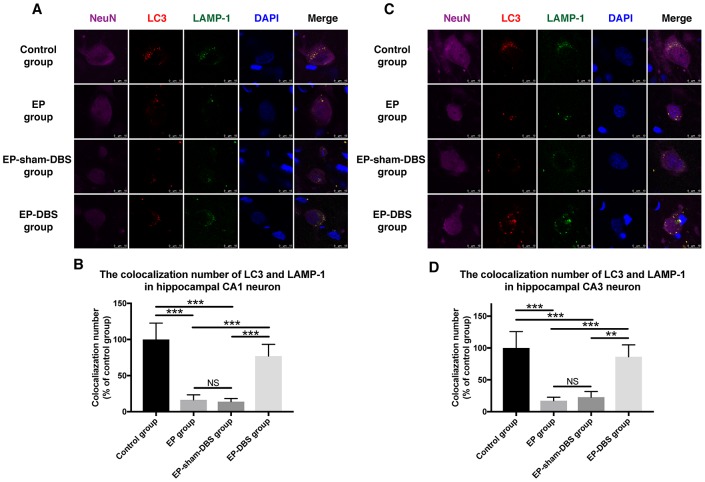
**ANT-DBS increased the colocalization of LC3 and LAMP1 in the hippocampal neurons of epileptic monkeys.** Colocalization of LC3 and LAMP-1 was decreased in hippocampal CA1 (**A**) and CA3 (**C**) neurons in EP and EP-sham-DBS groups, and increased by ANT-DBS. Colocalization number of LC3 and LAMP-1 in hippocampal CA1 (**B**) and CA3 (**D**) neurons. (n=3 in each group; in each monkey, a total of thirty cells in CA1 or CA3, the average immunofluorescent intensity of these cells was recorded) **P < 0.01; ***P < 0.001; NS, *P* > 0.05. Data were presented as mean ± SD.

We next measured the co-localization of LC3 and LAMP-1, a marker of lysosomes, in the hippocampus. Co-localization of the two proteins was decreased in hippocampal CA1 (F_(3,8)_= 26.604, *P* < 0.001) and CA3 (F_(3,8)_= 19.519, *P* < 0.001) neurons of the EP and EP-sham-DBS groups, but the situation was reversed in the EP-DBS group (Figure 7). Taken together, these results suggest that ANT-DBS stimulates autophagy by promoting autophagosome formation in epilepsy.

## DISCUSSION

**Figure 8:**
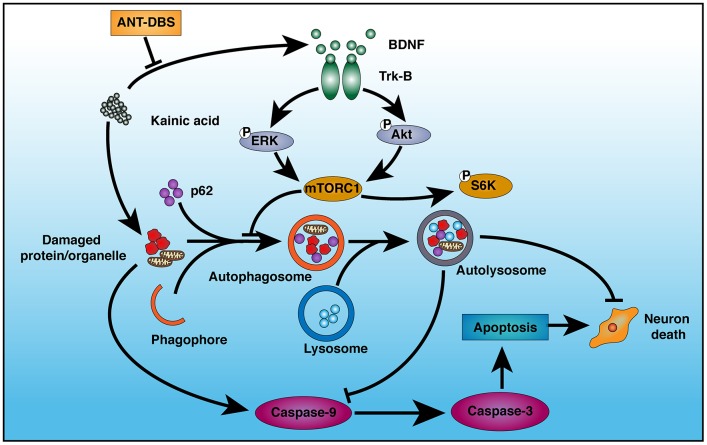
**Schematic illustration of the mechanism of ANT-DBS-mediated neuroprotection against KA-induced cell injury.** KA injures mitochondria, leading to induction of apoptosis via caspase-9 and -3. KA also inhibits autophagy by inducing BDNF expression and TrkB activation. ANT-DBS inhibits BDNF expression and TrkB activation induced by KA, followed by dephosphorylation of Akt and ERK, which lead to inactivation of mTORC1, thereby stimulating autophagy and clearance of injured cells by autolysosomes, and suppression of apoptosis.

We successfully established the NHP-TLE model, which was verified by behavior and SEEG monitoring, and studied the role of autophagy in the neuroprotective effects of ANT-DBS against KA-induced neuronal injury. The results showed that ANT-DBS-mediated hippocampal neurons protection in epileptic animals associated with increased autophagic activity which in turn was related to BDNF-TrkB signaling pathways and KA-induced apoptosis (Figure 8).

### NHP-TLE model and BDNF–TrkB signaling pathway

KA injection is one of the most reliable epilepsy models. Neuropathological and electroencephalographic characteristics observed in TLE patients can be observed in this model. It is also characterized by a latent period that follows the initial precipitating injury (i.e. status epilepticus) until the appearance of recurrent seizures, as observed in humans [27]. The status epilepticus and latent period following KA injection were obvious in our research.

In diverse animal models of TLE, seizures induce marked increases in BDNF expression and enhanced activation of TrkB in the hippocampus. Intrahippocampal infusion of BDNF and transgenic overexpression of BDNF or TrkB increase seizure susceptibility or severity, and conditional knockout of TrkB eliminates epileptogenesis altogether in the kindling model. Clinically, both BDNF and its receptor TrkB are elevated in patients with epilepsy, especially in the temporal and hippocampal area. Serum BDNF is frequently higher in patients with epilepsy and appears to be positively correlated with severity of the disease. Current evidence suggests that inhibiting BDNF–TrkB signaling represents a potential therapeutic strategy for epilepsy, especially for TLE [28]. In the present research, both BDNF and TrkB were increased in the hippocampus in the EP group compared with the control group. In contrast, expression of the two proteins was decreased in the hippocampus after ANT-DBS, which suggests that BDNF inhibition is involved in the neuroprotective mechanism of ANT-DBS. Therefore, ANT-DBS protects the neurons via the BDNF–TrkB signaling pathway and its downstream pathways.

One previous study found that BDNF–TrkB survival signaling underpins subthalamic nuclei (STN)-DBS-mediated protection of the dopamine neurons in the substantia nigra pars compacta in rats [29]. Another study found that STN-DBS increases BDNF in the substantia nigra reticulata, which suggests that the projected neurons themselves participate in STN-DBS-mediated regulation of BDNF [30]. The results from these studies differed completely from those from our study. The different targets and diseases might explain this difference. BDNF enhances excitatory synaptic transmission and reduces inhibitory synaptic transmission [31–33], the substantia nigra reticulata to thalamus are inhibitory projections. The BDNF inhibit this pathway and reduce the inhibitory output form the basal ganglia to the cortex [34]. This effect is beneficial in Parkinson’s disease, whereas, in epilepsy, reducing inhibitory synaptic transmission increases the probability of seizures.

Integrating all these evidences, a feasible hypothesis is that DBS regulates the biochemistry level in the circuits and leads circuits back into balance.

### BDNF-TrkB inhibition followed by autophagy activation contributes to the neuroprotective effect of ANT-DBS

Ligation of TrkB by BDNF leads to activation of distinct downstream MAPK/ERK and PI3K/Akt signaling pathways [35]. We evaluated these two pathways. Levels of both p-ERK and p-Akt decreased after stimulation, which suggests that both pathways are involved in the neuroprotective effect of ANT-DBS. Inhabitation of the two pathways further inhibit the mTORC1 [36]. Usually, the phosphorylation status of S6K can be used to evaluate mTORC1 activity [37]. So, we assessed activation of mTORC1 substrate S6K. p-S6K was decreased after ANT-DBS, implying inactivation of the upstream factor mTORC1 (a negative regulator of autophagy) [38], thereby activating autophagy.

Previous study has suggested LC3II was known as a marker of autophagosome [39]. In our study, LC3II protein level was increased in the EP-DBS group, which indicated an increase in autophagosome formation or decreased flux of autophagy. Inefficient flux of autophagy should result in an increase in the substrates, such as sequestosome-1 (SQSTM1)/P62, a polyubiquitin-binding protein [40]. On the contrary, P62 level decreased in the EP-DBS group compared that in the EP-sham-DBS and EP groups, suggesting the increased autophagosome formation by ANT-DBS, which might be related to BDNF. It has been found that BDNF inhibits 3-NP-induced autophagy via mTOR/c-Jun-dependent induction of p62 expression [41]. Therefore, these results indicate that ANT-DBS activates autophagy by inducing autophagosome formation. TLE has aberrant accumulation of damaged mitochondria, especially in the hippocampus, which may participate in epileptogenesis [42]. In addition to the mitochondria, many researches have confirmed that other organelles are also damaged in TLE [43]. Byproducts of protein, lipid and DNA oxidative damage also contribute to epileptogenesis [44]. All of these evidences indicate that accumulation of damaged proteins and organelles is related to epilepsy. Autophagy can eliminate the damaged proteins and organelles. In the present study, TEM showed that the ratio and severity of damaged mitochondria was reduced after ANT-DBS.

In this study, ANT-DBS not only led to activation of autophagy, but also inhibited apoptosis induced by KA. Autophagy and apoptosis are cellular housekeeping and tissue survival mechanisms. Autophagy involves the delivery of cytoplasmic contents and damaged organelles to the vacuoles or lysosomes for recycling or degradation [39]. An increase in mitochondrion-dependent apoptosis is observed in epilepsy [45]. The pivotal roles of apoptosis during hippocampal damage in KA-induced epilepsy are recognized [46]. Our results indicated that ANT-DBS suppressed cleaved-caspase-3/-9 expression induced by KA. This may be due to the positive effect of autophagy in eliminating the damaged proteins and organelles. Inhibition of autophagy accelerates apoptosis, whereas activation of autophagy has the opposite effect.

In conclusion, our study demonstrate that ANT-DBS may protect hippocampal neurons against apoptosis through activating autophagy, which was achieved by negatively regulating the BDNF–TrkB signaling pathway in an NHP-TLE model. Our findings provide insight into the molecular basis for the neuroprotective effects of ANT-DBS.

## MATERIALS AND METHODS

### Animals and ethics

Twenty-four male *rhesus monkeys* (mean ± standard deviation [SD] age: 7.3 ± 1.2 years; weight: 8.3 ± 1.4 kg), provided by the Laboratory Animal Center of the Military Medical Science Academy of China, were randomly assigned to the control group (n=6), EP (epilepsy) group (n=6), EP-sham-DBS group (n=6) and EP-DBS group (n=6). This study was approved by the Ethics Committee of Beijing Neurosurgical Institute (Process No. 201703002) and was consistent with the National Institutes of Health Guide for the Care and Use of Laboratory Animals (Publication No. 8023). Local anesthesia was applied with lidocaine injection (Shanghai Zhpharma co. ltd, Shanghai, China, 10mg/point) before incision. The application of narcotics (dosing interval) was based on the conscious state and suffering of the animals. When animals were sacrificed, ketamine was delivered to achieve deep anesthesia and to minimize suffering. Dexmedetomidine was used as an analgesic. Animals had free access to food and water, and excrement was removed daily and cages were cleaned weekly. Each monkey was raised in a single cage in an environmentally controlled room (23–25°C, 12-h light/12-h dark cycle, lights on at 07:00).

### Establishment of the EP model, behavior and stereoelectroencephalography (SEEG) monitoring

All monkeys achieved general anesthesia with intramuscular injection of Zoletil (5 mg/kg, Virbac, Alpes-Maritimes, France) and Dexdomitor (20 μg/kg, Zoetis, NJ, USA), before magnetic resonance image (MRI) (including 3D T1-, T2-weighted imaging and magnetic resonance angiography) with a 3-Tesla MRI scanner (SIGNA; GE Healthcare, Waukesha, WI, USA). The surgical planning of KA and normal saline injection was performed on the workstation of the neurosurgical robotic system (RM-100; Beijing Baihui Weikang Technology Co. Ltd., Beijing, China) based on preoperative MRI. The animals were placed on the stereotaxic instrument (model 1530; David Kopf Instruments, Tujunga, CA, USA) and rigidly fixed. In the EP, EP-sham-DBS and EP-DBS groups, KA (1 μg/μL; 1.5 μg/kg/target, 0.5 μl/min) was injected into the left hippocampus and amygdales according to the surgical planning (the microinjector was sustained for at least 5 min after injection), and normal saline (1.5 μl/kg/target) was injected into the same points in the control group. The vital signs were monitored throughout surgery.

SEEG electrodes were implanted into the left hippocampus at 24 h and 1 month after injection in order to detect epileptic discharge. Behavior of all animals was monitored based on the modified Racine scale as follows: 0, no response; I, facial movement; II, head nodding and absence; III, unilateral forelimb clonus; IV, bilateral forelimb clonus and rearing; and V, bilateral forelimb clonus and rearing and falling [47].

### DBS implantation and confirmation of lead location

Three weeks after KA injection, the EP-sham-DBS and EP-DBS groups received ANT-DBS implantation, which was similar to surgery in patients. The DBS leads (L301; Beijing PINS Medical Co. Ltd., Beijing, China) were designed to target the left ANT, according to individual MRI results and the atlas of the rhesus monkey brain [48]. Electrode implantation is conducted by the neurosurgical robotic system, this new method accuracy is confirmed by our previous study [26]. An extension was tunneled subcutaneously through the neck to the abdomen where the implantable pulse generator (IPG, G102; Beijing PINS Medical Co. Ltd., Beijing, China) was located. Postoperative computer tomography (CT) was conducted to evaluate complications and accuracy of lead placement. One week after lead implantation, the EP-DBS group was stimulated (1.5 V, 90 μs and 150 Hz; continuous stimulation), and stimulation contact was selected according to postoperative image fusion, and no electrical stimulation was delivered to the EP-sham-DBS group (Figure 1A).

### Tissue processing

Three months after KA and normal saline injection, all monkeys were deeply anesthetized (ketamine, 20 mg/kg, intramuscular injection) and sacrificed. Three animals in each group were randomly selected for fresh tissue processing (subgroup A, n=3), and the left hippocampus were removed and stored at −80°C. The remaining three monkeys in each group (subgroup B, n=3) were perfused with normal saline followed by 4% paraformaldehyde in 0.1 mol/L phosphate-buffered saline (PBS) until the neck was rigid and convulsions were observed. The brain was removed for pathological examination and divided into several 1cm-thickness coronary sections via a self-developed device [26]. Then, the 1cm-thickness coronary section was dehydrated in 20% and 30% sucrose solution and cut on a cryostat at a thickness of 20 μm into sections.

### Western blotting

Brain tissue was washed with ice-cold PBS and lysed in radioimmunoprecipitation assay buffer comprising 50 mM Tris–HCl (pH 7.4), 150 mM sodium chloride, 1% Nonidet P-40, and 0.1% sodium dodecyl sulfate (SDS) containing sodium phosphate and protease inhibitor cocktails. Homogenates were centrifuged at 12,000 × g for 20 min. The protein concentration in the supernatant was determined with a bicinchoninic acid protein assay kit (Pierce, Rockford, IL, USA). A total of 60 μg protein per sample was resolved by SDS-PAGE on a 12% polyacrylamide gel and the protein bands were transferred to a polyvinylidene difluoride membrane (Millipore, Billerica, MA, USA). After blocking with 10% milk for 1 h, the membrane was incubated with the following primary antibodies: rabbit anti-β-actin (1:10000, A5060; Sigma–Aldrich, St. Louis, MO, USA), mouse anti-NeuN (1:1000, MAB377; Millipore), rabbit anti-phosphorylated (p)-Akt (1:1000, 4060S; Cell Signaling Technology, Danvers, MA, USA), rabbit anti-Akt (1:1000, 4691S; Cell Signaling Technology), rabbit anti-p-Erk (1:1000, 9101S; Cell Signaling Technology), rabbit anti-Erk (1:1000, 4695S; Cell Signaling Technology), rabbit anti-p-S6K (1:1000, PA5-17884; ThermoFisher Scientific, Schwerte, Germany), rabbit anti-S6K (1:1000, 2708S; Cell Signaling Technology), rabbit anti-light chain 3 (LC3) (1:1000, L7543; Sigma–Aldrich), mouse anti-P62 (1:1000, ab56416; Abcam, Cambridge, MA, USA), rabbit anti-BDNF (1:1000, NBP1-59304; Novus Biologicals, Littleton, CO, USA), mouse anti-TrkB (1:500, NPB1-47898; Novus Biologicals), rabbit anti-cleaved caspase-3 (1:1000, 9661; Cell Signaling Technology), rabbit anti-caspase-3 (1:1000, 9662; Cell Signaling Technology), rabbit anti-cleaved caspase-9 (1:1000, 9507; Cell Signaling Technology), and mouse anti-caspase-9 (1:1000, 9508; Cell Signaling Technology). Membranes were incubated with secondary antibody (1:10000, LI-COR, Lincoln, NE, USA) and protein bands were visualized by enhanced chemiluminescence and quantified using ImageJ software (US National Institutes of Health).

### Evaluation of protein colocalization

Brain tissue sections were rinsed in PBS and permeabilized with 0.3% Triton X-100 in PBS for 10 min at room temperature. After blocking with 10% normal goat serum for 1 h, sections were incubated overnight at 4°C with rabbit anti-NeuN (1:500, ab128886; Abcam), mouse anti-LC3 (1:100, M152–3; MBL), and rat anti-lysosomal-associated membrane protein (LAMP)1 (1:50, sc-19,992; Santa Cruz Biotechnology, Santa Cruz, CA, USA) antibodies, followed by Alexa Fluor 647-, 594-, or 488-conjugated secondary antibody (1:500; Thermo Fisher Scientific; Massachusetts; USA) for 1 h at room temperature. Cell nuclei were visualized by counterstaining with 4′,6-diamidino-2-phenylindole (DAPI) (Sigma–Aldrich). Sections were mounted on slides with 70% glycerol and covered with coverslips, and were visualized on a confocal microscope (TCS SP8; Leica, Solms, Germany). Five sections (distance between each section was 1mm) in hippocampus head were chosen from each monkey, and six cells in hippocampus CA1 or CA3 were randomly selected in each section. A total of thirty cells in hippocampal CA1 or CA3 were performed for evaluation in each monkey. Immunofluorescent intensity was measured via the ratio of cell expression to background in each view.

### Transmission electron microscopy (TEM)

TEM was performed according to a previous study [25]. The hippocampus was washed in 0.1 M PBS and stored in 2.5% glutaraldehyde in 0.1 M PBS until processed. The slices were washed in 0.1 M PBS, post-fixed in 1% osmium tetroxide in 0.1 M PBS for 2 h and again washed in 0.1 M PBS. Ultrathin sections were observed using an electron microscope (JEM2100; JEOL, Japan), and were observed in twenty random cells (neuronal injury, autophagosome) and visual fields (mitochondrial injury) from each sample, scored according to the criteria described below, and recorded, as described previously [49, 50]. When the condition was considered to fall between two scores, an increment of 0.5 point was added to the score. If more than one neuron or mitochondrion was observed in one field, the average grade was recorded.

### Statistical analysis

All data were presented as mean ± standard deviation (SD) and analyzed by independent-samples T test and one-way ANOVA followed by least significant difference (LSD) test for multiple comparisons. Statistical data analysis was performed using SPSS version 21.0 software (IBM, Armonk, NJ, USA). A significant difference was confirmed between groups when *P* was ≤0.05. All data were plotted using GraphPad Prism version 7.0 (GraphPad Software, La Jolla, CA, USA).
